# Cellular Basis of Tissue Regeneration by Omentum

**DOI:** 10.1371/journal.pone.0038368

**Published:** 2012-06-06

**Authors:** Shivanee Shah, Erin Lowery, Rudolf K. Braun, Alicia Martin, Nick Huang, Melissa Medina, Periannan Sethupathi, Yoichi Seki, Mariko Takami, Kathryn Byrne, Christopher Wigfield, Robert B. Love, Makio Iwashima

**Affiliations:** 1 Department of Microbiology and Immunology, Stritch School of Medicine, Loyola University Chicago, Chicago, Illinois, United States of America; 2 Department of Medicine, Stritch School of Medicine, Loyola University Chicago, Chicago, Illinois, United States of America; 3 Department of Thoracic and Cardiovascular Surgery, Stritch School of Medicine, Loyola University Chicago, Chicago, Illinois, United States of America; Instituto Butantan, Brazil

## Abstract

The omentum is a sheet-like tissue attached to the greater curvature of the stomach and contains secondary lymphoid organs called milky spots. The omentum has been used for its healing potential for over 100 years by transposing the omental pedicle to injured organs (omental transposition), but the mechanism by which omentum helps the healing process of damaged tissues is not well understood. Omental transposition promotes expansion of pancreatic islets, hepatocytes, embryonic kidney, and neurons. Omental cells (OCs) can be activated by foreign bodies *in vivo*. Once activated, they become a rich source for growth factors and express pluripotent stem cell markers. Moreover, OCs become engrafted in injured tissues suggesting that they might function as stem cells.

Omentum consists of a variety of phenotypically and functionally distinctive cells. To understand the mechanism of tissue repair support by the omentum in more detail, we analyzed the cell subsets derived from the omentum on immune and inflammatory responses. Our data demonstrate that the omentum contains at least two groups of cells that support tissue repair, immunomodulatory myeloid derived suppressor cells and omnipotent stem cells that are indistinguishable from mesenchymal stem cells. Based on these data, we propose that the omentum is a designated organ for tissue repair and healing in response to foreign invasion and tissue damage.

## Introduction

The healing potential of the omentum has been utilized clinically by transposing the omental pedicle or flap to injured organs (omental transposition) for many decades [1]–[3]. It is located in the peritoneal cavity and is known for its diverse functions of controlling the spread of inflammation, and promoting revascularization, reconstruction and tissue regeneration [4], [5]. Omental transposition has been used to treat infections such as mediastinitis and chronic cranial osteomyelitis. In addition, it has been used in brain surgeries [6]–[9], for revascularization of ischemic brain and myocardium, lower and upper extremities [10]–[12], and for reconstruction of head and neck defects [13], [14]. Further, the omentum has also been used to support regeneration of neurons across a freshly transected spinal cord in experiments in cats and also in one patient [15]–[17] resulting in the unexpected recovery of limb function. The production of a variety of growth factors by the omentum provides a possibility to sustain transplanted pancreatic islets, expand cultured hepatocytes and regenerate liver, and grow embryonic kidney and pancreas anlagen into adult organs [18]–[22].

Despite reports of the benefits of omental transposition in acute injury, studies addressing the mechanism by which the omentum exerts such effects are lacking. Recently investigators have shown that the omentum can be activated in the presence of foreign bodies injected in the peritoneal cavity of rats. Once activated, the flimsy sheet like organ enlarges in size and mass wherein the new cells are predominantly non-fat stromal cells [23], [24]. These stromal cells are a rich source of growth factors like fibroblast growth factor (FGB) and vascular endothelial growth factor (VEGF) and express adult stem cell markers including SDF-1α, CXCR4, WT-1, as well as pluripotent embryonic stem cell markers, Nanog, Oct-4, and SSEA-1 [23], [25], [26]. Further, they were engrafted in injured tissues showing that they function as stem cells *in vivo* [23], [25]. Together, the data suggest that the omentum contains the potent ability of tissue regeneration and may be useful for treatment for various types of diseases involving tissue damages.

In this study, we further characterized the anti-inflammatory and tissue healing properties of cells derived from the omentum. We determined that there are multiple subsets of cells that accumulate in the activated omentum with different roles in attenuating inflammation and supporting tissue regeneration.

## Results

**Figure 1:**
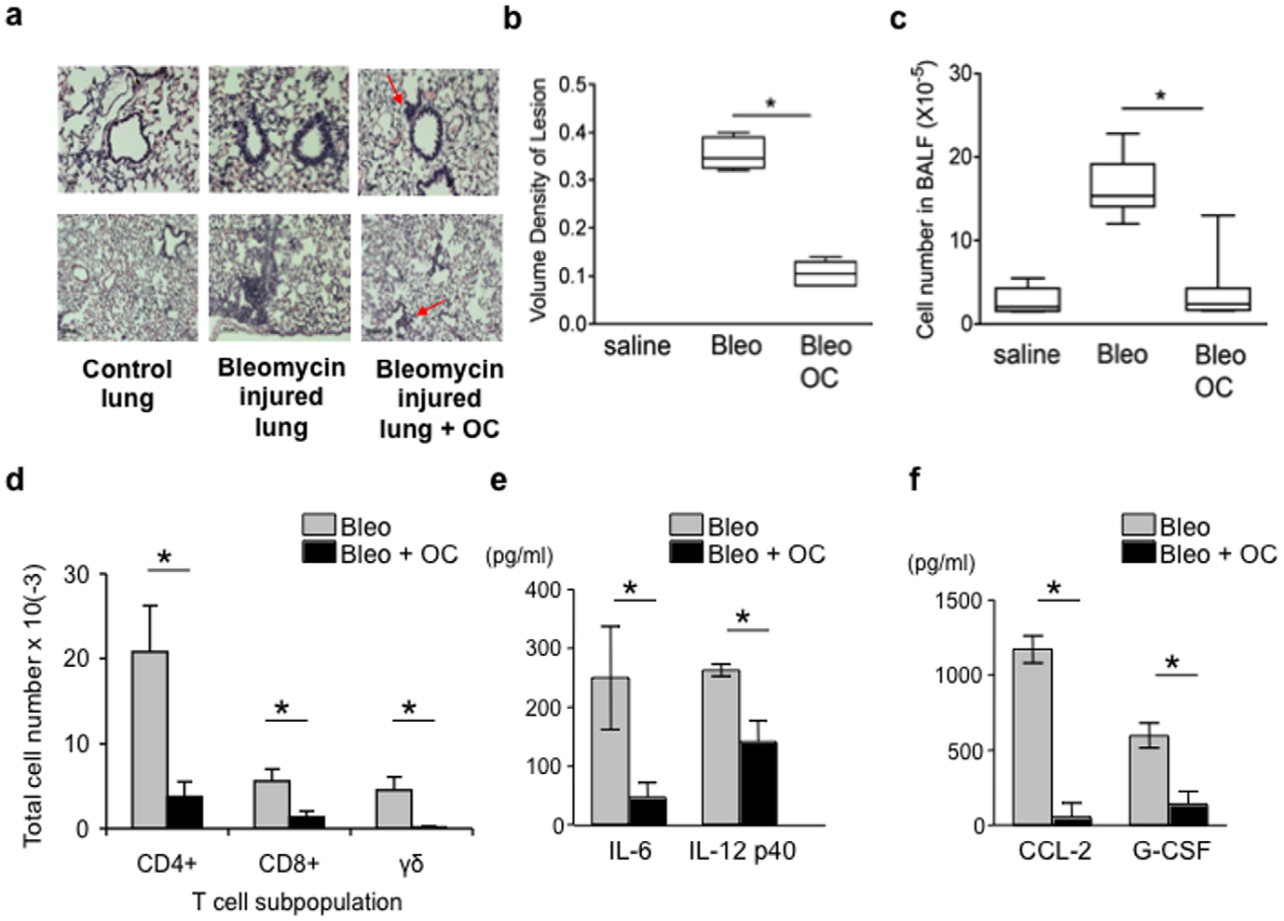
Effect of omentum cells on inflammation in the bleomycin induced mouse lung injury model. Saline or bleomycin was given intra-tracheally to C57BL/6 mice. 4 hours later, mice received i.p. injections of omentum cells (1×10^6^ cells) or saline alone. (**a**) H&E staining of lung sections from mice sacrificed 7 days after bleomycin injury. Upper panels (×10), Lower panels (×20). (**b**) Quantification of inflammation by a volume density of lesion analysis. (**c, d**) Total cell numbers and percentages of T cell subsets in the bronchoalveolar lavage fluid (BAL) 7 days after bleomycin instillation. (**e, f**) Cytokine analysis on BAL samples 3 days after bleomycin instillation. * denotes for p<0.05.

We tested the effect of omentum cells in tissue repair by using an acute lung injury model with intratracheal (IT) injection of bleomycin [27]. To collect sufficient numbers of omentum cells, the omentum was expanded in mice by intraperitoneal (i.p.) injection of polyacrylamide beads as reported for rats [23]. Seven days after injection, both the greater and lesser omentum were expanded in mice as in rats (Fig. S1). Single cell suspensions were made from expanded omentum, and cells were adoptively transferred into mice that were administered with bleomycin (Fig. 1). Compared to mice injected with bleomycin alone, mice that received adoptive transfer of omentum cells showed a significant reduction in the level of tissue inflammation and cellular accumulation in the lung (Fig. 1a). Only a few inflammatory areas (arrows) in the parenchyma were apparent. Morphometric analysis by volume density of lesion and the number of cells detected in bronchoalveolar lavage fluid (BAL) revealed a significant reduction in the extent of inflammation in the lung (Fig. 1b and c). All T cell subsets, CD4, CD8, and γδ T cells in BAL were reduced (Fig. 1d). Cytokine profiles in BAL showed that injection of omentum cells reduced the levels of pro-inflammatory cytokines such as IL-6 and IL-12 p40 (Fig. 1e). In addition, the concentration of CCL2 (MCP-1) and G-CSF was significantly reduced in the omentum cell-treated group compared to the bleomycin only group (Fig. 1f). Together, the data suggest that transferred omentum ameliorates bleomycin-induced lung injury either by reduction of immune/inflammatory responses and/or accelerated recovery of the damaged lung.

**Figure 2:**
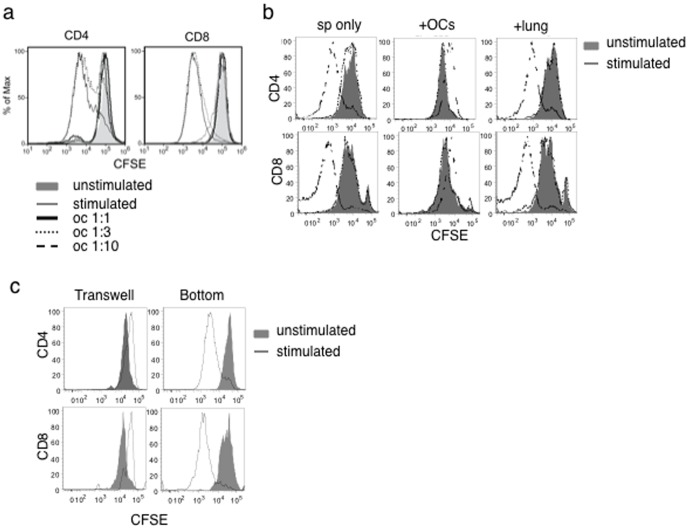
Dose- and cell-cell contact-dependent suppression of T cell proliferation by omentum cells. (**a**) CFSE labeled splenocytes were cultured with omentum cells at different ratios of omentum cells with or without 1 µg/ml anti-CD3. Cells were labeled with anti-CD4 or anti-CD8 and analyzed by flow cytometry. Omentum cells were added at different ratios to splenocytes (1∶1, 1∶3, or 1∶10) as indicated in the figure. (**b**) CFSE labeled splenocytes were cultured with omentum cells or lung cells in a 1∶1 ratio and surface labeled as in (**a**). (**c**) CFSE labeled splenocytes were cultured in the transwell with or without anti-CD3 across a semi-permeable membrane containing omentum cells and CFSE labeled splenocytes.

**Figure 3:**
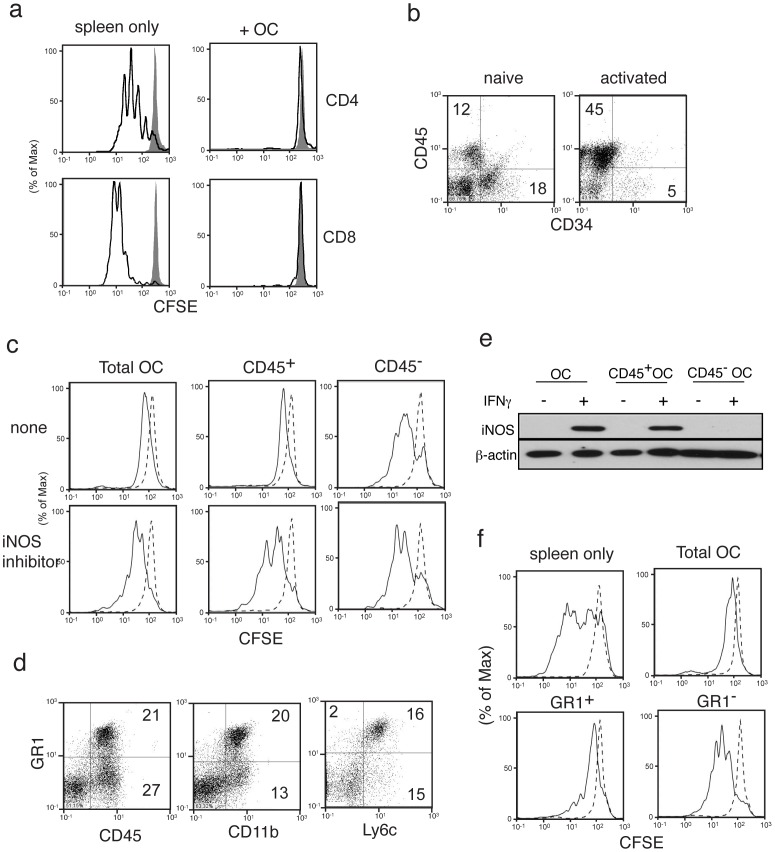
Effect of omentum cells on *ex vivo* T cell proliferation. (**a**) CFSE labeled splenocytes were cultured with or without omentum cells in a 1∶1 ratio for 3 days. T cells were either left unstimulated (grey solid) or stimulated with 1 µg/ml anti-CD3 (dark lines). Cells were labeled with anti-CD4 and analyzed by flow cytometry. (**b**) Surface phenotypic analysis of cells derived from naïve and day 7 omentum by flow cytometry. (**c**) Omentum cells were sorted into CD45^−^ and CD45^+^ cells before culturing with CFSE labeled splenocytes with 1 µg/ml anti-CD3 (solid lines) or left unstimulated (dotted lines). Cells were harvested and surface labeled with anti-CD4. (**d**) Surface antigen expression by CD45^+^ omentum cells: Activated omentum cells were stained and analyzed for the expression of antigens indicated. (**e**) iNOS expression was determined in total omentum cells, CD45^+^ omentum cells, or CD45^−^ omentum cells upon IFNγ stimulation for 24 hrs by western blotting. (**f**) Omentum cells were sorted into Gr1^+^/Gr1^−^ cells and tested as in (c) for the effect on T cell proliferation.

T cells play critical roles in inflammation and following fibrosis in the bleomycin-induced tissue injury models [28]–[30]. To elucidate the mechanism by which omentum cells helped healing of the lung, we determined if omentum cells suppress T cell proliferation and function. CFSE labeled splenocytes were stimulated by anti-CD3 antibody to induce proliferation in the presence or absence of omentum cells. After 3 days, the proliferation of CD4^+^ and CD8^+^ T cells was dramatically reduced in the presence of omentum cells (Fig. 2a and Fig. 3a). Inhibition of T cell proliferation was observed when omentum cells were added as low as 1/10 of splenocytes and correlated to the omentum cell numbers added to the culture (omentum∶splenocytes ratios ranged from 1∶10∼1∶1) (Fig. 2a). Inhibition of cell proliferation was not due to the deterioration of culture conditions since addition of the same number of suspended lung cells had no effect on T cell proliferation (Fig. 2b). Further, inhibition of T cell proliferation by omentum cells was not observed when cells were separated in a transwell culture system (Fig. 2c) suggesting that cell-cell contact or locally acting factors are important for T cell suppression.

These data suggest that the omentum is a potent immuno-regulatory organ, and not what has been postulated as a cluster of adipocytes associated with secondary lymphoid organs [31]. We therefore compared surface antigen expressions by activated and naïve omentum cells, because only activated omentum cells suppressed proliferation of T cells (data not shown). Previous reports showed the presence of cells with stem cell functions in the omentum [24], [25]. Mesenchymal stem cells (MSCs) known to have immunoregulatory functions, have been characterized as CD45−Cd34+ in C57BL.6 mice [32]. Thus, we analyzed CD45 and CD34 expression by the omentum and identified three subsets of cells in naïve omentum: CD45^−^CD34^+^, CD45^+^CD34^−^, and CD34^−^CD45^−^ cells (Fig. 3b). In activated omentum, CD45^+^CD34^−^ cells were substantially increased (Fig. 3b). Few, if any lymphocytes were found in either naïve or activated omentum (data not shown). Since increase of CD45^+^ cell number and suppression of T cells by omentum cells correlated positively, we sorted omentum cells into CD45^+^ and CD45^−^ cells and determined which subset suppresses T cell activation. When separated, CD45^+^ omentum cells suppressed splenic T cell proliferation as effectively as whole omentum cells while CD45^−^ cells did not (Fig. 3c).

Recent reports have identified an immunoregulatory CD45^+^ cell subset called myeloid derived suppressor cells (MDSCs) [33]. MDSCs consist of a mixture of cells including granulocytes, monocytes and dendritic cells and are defined in mice on the basis of CD11b and Gr1 expression and their ability to suppress T cell proliferation [34], [35]. Among activated omentum cells, approximately 50% of CD45^+^ cells were Gr1^+^ and CD11b^+^ (Fig. 3d). MDSCs are further divided into polymorphonuclear (PMN-MDSCs) or mononuclear (MO-MDSCs) MDSCs [33]. PMN-MDSCs express low or intermediate Ly6c while MO-MDSCs are Ly6c^high^. Data show that 16% of omentum cells are Gr1^+^Ly6c^+^ while only 2% is GR1^+^ Ly6c^−^, thus a majority of Gr1^+^ cells from omentum are Ly6c^high^, suggesting that they are MO-MDSCs (Fig. 3d). MO-MDSCs are known to suppress T cell proliferation in an inducible nitric oxide synthase (iNOS) dependent manner dependent in response to IFNγ exposure [36], [37]. Indeed, CD45^+^, but not CD45^−^, omentum cells express iNOS when treated with IFNγ (Fig. 3e). Moreover, an inhibitor for iNOS (L-NAME) abrogated suppression of T cell proliferation by CD45^+^ omentum cells (Fig. 3c). Addition of anti-IFNγ antibody also abrogated T cell suppression by omentum cells (not shown). Based on these data, we hypothesized that suppression of splenic T cells by omentum cells is mediated by MO-MDSCs. Indeed, when we separated omentum cells by Gr1 expression, Gr1^+^ cells but not Gr1^−^ cells, suppressed T cell proliferation (Fig. 3f).

**Figure 4:**
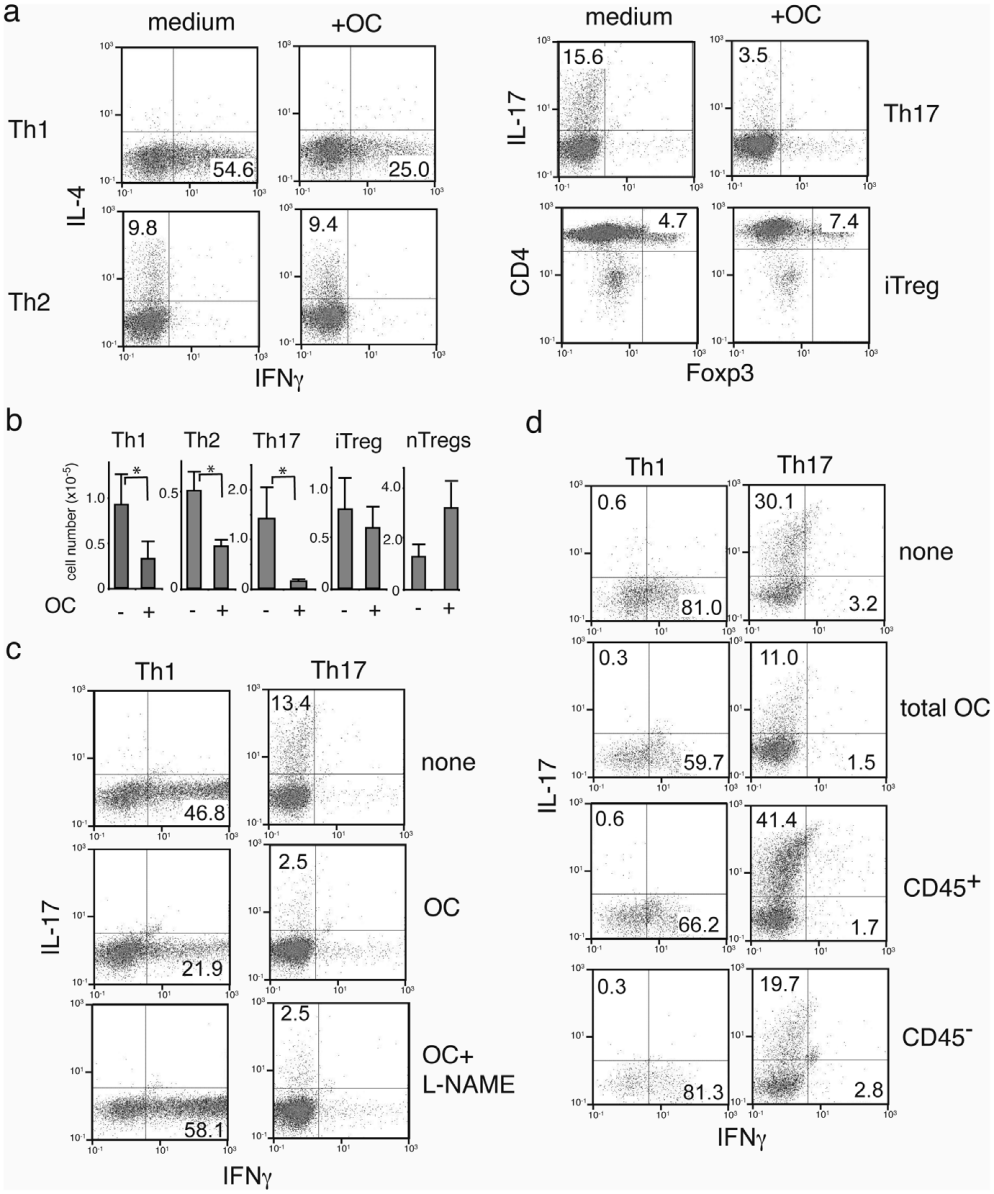
Suppression of effector but not regulatory T cells by omentum cells. (**a**) Immunosuppressive functions of omentum cells on effector T cells. Naïve CD4^+^ T cells were induced to differentiate into Th1, Th2, Th17, or iTregs. 5 days after induction, omentum cells were added to each group of cells. T cells were maintained further with the same culture medium for 2 days, harvested, and were stimulated with PMA and ionomycin for 4 hours to induce cytokine production. (**b**) Cells were cultured with (+) or without (−) omentum cells and treated as in (a). Cell numbers that are expressing IFNγ, IL-4, IL-17, or Foxp3 were determined after intracellular cytokine stain. For nTregs, CD4^+^CD25^+^ from spleen were expanded prior to co-culture for 2 weeks, then cultured with omentum cells for 2 days. (**c**) Effect of iNOS inhibitor on Th1 and Th17 inhibition by omentum cells. Differentiated Th1 or Th17 cells were cultured with omentum cells in the presence/absence of an iNOS inhibitor for 2 days as in (a). (**d**) Omentum cells were sorted into CD45^−^ and CD45^+^ cells, then co-cultured with CD4^+^T cell differentiated into Th1 (upper panels) or Th17 (lower panels) cells for 5 days. After 2 days of co-culture, cells were harvested and cytokine profiles were determined as in (a).

Adoptive transfer of omentum cells imposed a profound effect on bleomycin-induced lung inflammation. Recent studies revealed the significance of Th17 cells in this disease model [38]. Thus, we tested if omentum cells also inhibit activation/proliferation of already differentiated effector/regulatory T cells. When naive CD4^+^ T cells were differentiated into effector/regulatory T cells *in vitro*, and then co-cultured with omentum cells for 5 days, a significant reduction in cell numbers and expression of IFNγ (Th1 cells) and IL-17 (Th17 cells) was found (Fig. 4a, b). Little effect on Th2 cells was observed. Importantly, no effect on Foxp3 expression or numbers of Foxp3^+^ iTreg cells was detectable. Spleen-derived nTregs showed a mild increase in cell number when co-cultured with omentum cells. Together, the data show that omentum cells have cell type specific inhibition on effector type T cells.

As we observed for total splenic T cells, suppression of Th1 cells by omentum cells was iNOS dependent since addition of an iNOS inhibitor lead to a significant increase in the frequency of IFNγ^+^ cells (21.9% vs. 58.1%). In a clear contrast, the frequency of omentum-treated IL-17^+^ cells did not change between carrier control samples and the iNOS inhibitor treated samples (2.5% and 2.5%, respectively) (Fig. 4c). Moreover, while CD45^+^ omentum cells are responsible for suppression of naïve T cells and Th1 cells (Fig. 4d), they showed little, if any, effect on Th17 cells. In contrast, CD45^−^ subset of omentum cells reduced IL-17^+^ cell activation and expansion. Together, the data demonstrate that there are CD45^−^ immunoregulatory cells present in the omentum that block Th17 cells in an iNOS independent manner.

**Figure 5:**
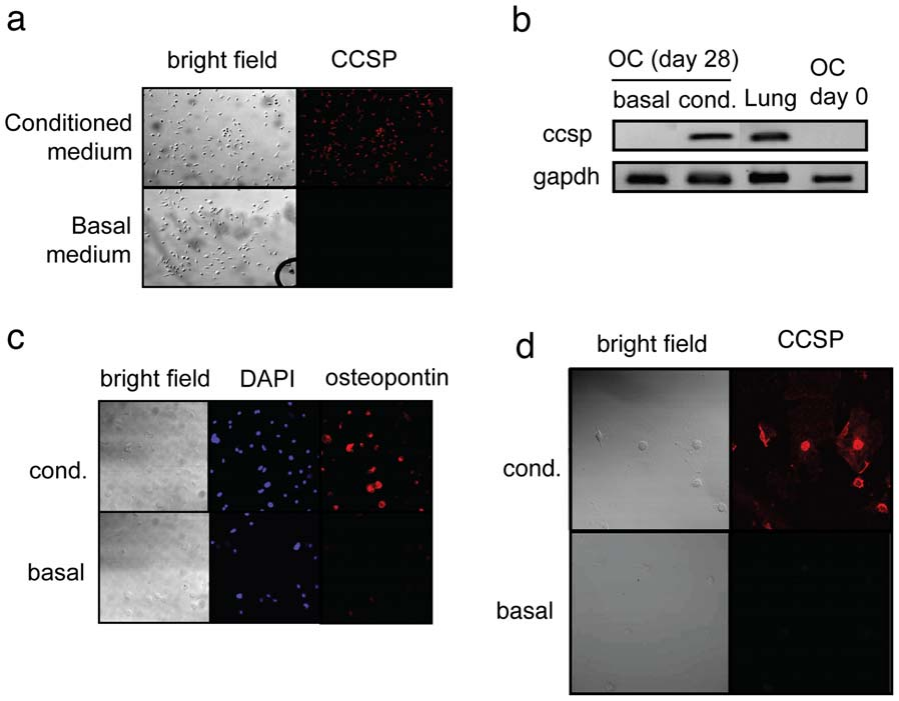
Differentiation of omentum cells into lung epithelium or osteoblasts. Omentum cells were cultured in the medium conditioned to induce (a, b, d) lung epithelium or (c) osteoblast. (**a**) After 5 weeks of culture, cells were stained for expression of CCSP. (×10) (**b**) Cells cultured as in (a) were used to determine the expression of *ccsp* mRNA by semi-quantitative RT-PCR. *gapdh* mRNA level was examined to determine the amount of mRNA in each sample. (**c**) Omentum cells cultured in the basal (lower panels) or medium conditioned for osteoblast induction (upper panels) for 2 weeks. Cells were stained by DAPI (middle) or with anti-osteopontin antibody. (**d**) Omentum cells were separated into CD45^+^, CD45^−^CD34^+^, or CD45^−^CD34^−^ cells, and were cultured in the medium conditioned for lung epithelium cell induction. Only CD45^−^CD34^+^ cells survived and expanded to the analyzable level in the conditioned medium and are shown.

**Figure 6:**
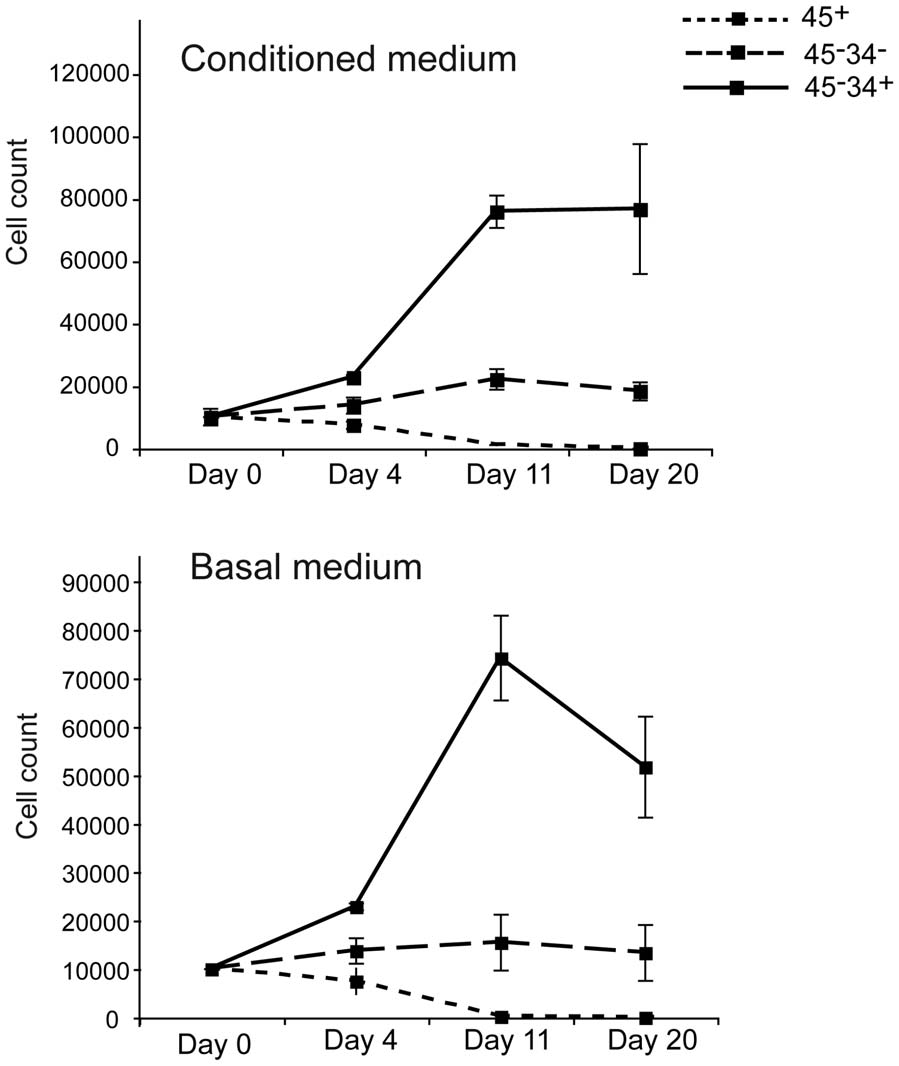
Survival and expansion of omentum cell subsets *ex vivo*. Omentum cells were separated into CD45^+^, CD45^−^CD34^+^, and CD45^−^CD34^−^ subsets and cultured in the conditioned medium for lung epithelium differentiation (upper panel) or in the basal medium. Cell numbers for each group were counted on days as indicated in triplicate.

Amelioration of bleomycin-induced lung injury by adoptive transfer of omentum cells could also be promoted by potentiating tissue regeneration processes. Previous reports suggest that rat omentum contains cells that have stem cell functions to differentiate into nerve cells, adipocytes, and hepatocytes [39]–[41]. To determine if mouse omentum contains stem cells, we cultured omentum cells in medium conditioned to induce differentiation into lung epithelial cells [42], [43]. After 28 days, we determined expression of a lung specific antigen, clara cell secretory protein (CCSP). Cells maintained in the lung epithelial-inducing medium clearly expressed CCSP detected both by immunofluorescent analysis and by RT-PCR (Fig. 5a, b). We also cultured omentum cells in medium inducing differentiation into osteoblasts [44]. 2 weeks after the start of culture, we observed a high level of osteopontin expression (Fig. 5c). The data show that a subset of omentum cells is omnipotent in differentiation. To determine the phenotype of the stem cell subset, we separated omentum cells into CD45^+^ and CD45^−^ subsets. A previous report showed that bone marrow derived MSCs from C57BL/6 mice are CD45^−^CD34^+^, although CD34^+^ cells are considered to be hematopoietic stem cells in other strains of mice and in humans [32]. Thus, we further separated CD45^−^ cells into CD34^+^ and CD34^−^ subset. In the lung epithelial-inducing medium or in basal medium, CD45^+^ cells died within 2 weeks of culture. CD45^−^CD34^−^ cells survived the culture but showed no expansion. Cells derived from CD45^−^CD34^+^ cells expanded under these conditions and differentiated into CCSP^+^ cells (Fig. 6, Fig. 5d). Together, the data show that CD45^−^CD34^+^ cells are the major source of stem cells from activated omentum.

**Figure 7:**
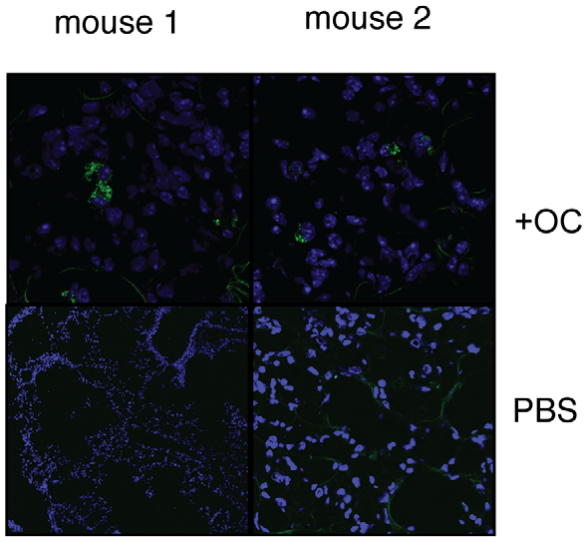
Presence of omentum-derived cells in the bleomycin-injured mouse lung. Omentum cells from transgenic mice that express GFP in non-hematopoietic cells or PBS (Bleo only) were injected into mice that underwent bleomycin-induced lung injury. 1 week after cell injection, mice were analyzed for the presence of GFP^+^ cells (green cells). (×10).

Lastly, we tested if omentum cells were integrated into lung tissues when injected into bleomycin-injected animals. To test this, we used omentum cells from transgenic mice that express GFP in a tissue non-specific manner [45]. When we adoptively transferred GFP^+^ omentum cells into bleomycin-injected animals, we detect the presence of GFP^+^ cells in the lung of bleomycin-injected animals (Fig. 7). Thus, a subset of the omentum cells, either as hematopoietic cells or lung epithelium cells, can migrate into the lung tissue.

## Discussion

Identified as the ‘policeman’ of the abdomen over a hundred years ago [46], the omentum has since been used clinically for its ability to seek and contain the site of injury. While expression of stem cell markers and angiogenic growth factors have been identified to explain some of its regenerative and revascularization properties, little is understood about the mechanisms of its anti-inflammatory and wound healing properties. Here we demonstrated that activated omentum has at least three functionally distinct groups of cells that can facilitate regeneration of damaged tissue: immunomodulatory CD45^+^ Gr1^+^ MDSCs; CD45^−^ cells that have the ability to suppress Th17 cells; and CD45^−^CD34^+^ cells that show MSC-type stemness (???). A striking feature of omentum is that, unlike secondary lymphoid organs such as lymph nodes or spleen, it enlarges in response to foreign objects and acquires a large number of immunomodulatory cells along with cells with stem cell function. This type of response has not been recognized for any other organ and is quite unique to the omentum. The data reported here reveal the cellular subsets that are recruited to the omentum and explain how the omentum provides support for tissue healing/regeneration as observed by many in clinical setting.

Naïve omentum consists of adipocytes with some ‘milky spots’ predominantly consisting of cells characterized as macrophages [47]. Upon activation with foreign bodies, the non-adipose areas expand, with a dramatic increase in the percentage of CD45^+^ cells within the omentum cell population. A subset of CD45^+^ cells suppress naïve CD4^+^ and CD8^+^ T cell and Th1 cell proliferation in an iNOS dependent manner. Surface antigen expression of CD11b and Gr1 by these cells suggests that these cells are MDSCs [34]. MDSCs play important roles in suppressing airway inflammation [48], suggesting MDSCs may be the cells responsible for inhibiting inflammation in our lung injury model.

Activated omentum also contain cells with stem cell functions among the CD45^−^CD34^+^ subset. Surface antigen expression matches that of MSCs, which have potential to differentiate into bone, fat and cartilage [49], [50]. Recent studies have shown that MSCs not only differentiate into cells of mesodermal lineage, but also cells of endodermal and ectodermal lineages, including cardiomyocytes [51], [52], lung epithelial cells [53]–[55], hepatocytes [56], [57], neurons [58], [59], and pancreatic islets [60], [61]. MSCs were originally identified from bone marrow (BM) but cells with similar characteristics have been isolated from other mesodermal tissues such as adipose tissue [62]. MSCs secrete a variety of factors including those with immunosuppressive functions [63] and provide a regenerative microenvironment for injured tissues to limit the area of damage and to mount a regenerative response [64]. Our data suggest that the presence of functional MSCs is a part of the mechanism by which the omentum imposes tissue healing support on the damaged tissues.

The omentum also contains CD45^−^ cells that inhibit Th17 cells. The effect is independent of iNOS. Currently, it is not clear whether this function is derived from MSCs. The effect of MSCs on Th17 is controversial [34], [35], [36] and will require further investigation.

To our knowledge, this is the first demonstration that shows co-localization of MSCs and MDSCs in a normal tissue in a large scale. Co-presence of these two groups of cells provides an explanation for why omentum has the ability to support healing of damaged tissues. Cells that expand in the omentum, mainly MDSCs, are regulatory toward inflammatory and immune responses, and MSCs can function as the source of trophic activity. How these immunomodulatory cells and stem cells are recruited and function together in the omentum is a question of significant implications in clinical applications since elucidation of the mechanism will lead to development of effective methods for tissue regeneration and repair. Whether these MDSCs and MSCs originate from the omentum or other tissues is currently under investigation. MDSCs accumulate in tumors and participate in the suppression of immune responses to tumor antigens [34]. In addition, MDSCs also expand in acute and chronic inﬂammatory sites [65]. In contrast, MSCs are known to be adherent and fibroblast-like from their *in vitro* observations and how they migrate and/or expand *in vivo* is not well understood [66]. In this manuscript, omentum activation was carried out by injection of polyacrylamide beads. Polyacrylamide microcapsule has been tested for biocompatibility and was found mildly inflammatory but did not provoke significant proliferation of immune cells [67]. Polyacrylamide gel was initially infiltrated by macrophage and slow integrated within its host tissue via a thin fibrous network [68]. It is considered biologically inert and used as a tissue filler. How the omentum recognizes polyacrylamide beads is not clear, but it is well known that the omentum responds to foreign non-infectious objects such as catheter tubing in patients during peritoneal dialysis [69]. Either MDSCs or other phagocytic cells may be the initial group of cells that encounter the beads and expand and/or recruited to the omentum. After this, MSCs within the omentum might expand and/or are recruited from other tissues.

Together, these data demonstrate that the omentum is equipped to help tissue healing, as observed clinically in various settings, and that the function results from multiple subsets of cells that specialize in immune modulation and tissue regeneration. In the future, cells from the omentum may be applicable for use in support for tissue healing and regeneration in a variety of inflammatory disorders. Recent advances in stem cell research have also highlighted the role played by such cells and their microenvironment termed the stem cell niche where tissue renewal takes place. Understanding of how to provide the proper niche for stem cells is critical information for regenerative medicine and determination of how the omentum works could provide some physiological answers to this profound question.

## Materials and Methods

### Ethics Statement

All experiments were performed in accordance with the guidelines and under approval of the IACUC of Loyola University Chicago Stritch School of Medicine (#2009073).

### Mice

C57BL/6 mice at 8–10 weeks were obtained from Harlan Laboratory (Madison, WI). All procedures were reviewed and approved by the Institutional Animal Care and Use Committee (IACUC) of Loyola University, Chicago.

### Isolation of cells from the omentum

C57BL/6 mice were injected intraperitoneally with 1 ml of polyacrylamide bead slurry (Bio-gel P-60, Biorad Laboratories, Hercules, CA, 1∶1 in 1× PBS). Mice were sacrificed at day 7 and the omentum harvested. Omentum cells were isolated using a modified method from Garcia-Gomez et al [70]. Briefly, omentum was chopped into small pieces and digested in 1 mg/ml collagenase type I (Sigma, St. Louis, MO) for 30 minutes at 37°C. Cells were then washed and separated over a ficoll gradient. Cells from the interface were passed through a cell strainer and surface stained for phenotypic analysis or put into culture in complete RPMI media (RPMI 1640 medium supplemented with 10% FCS (Atlanta Biologicals), beta-mercaptoethanol (50 µM), glutamine, sodium pyruvate (1 mM), and amino acids (Invitrogen).

### Cell culture

Splenocytes from C57BL/6 mice were labeled with 2 µM CFSE and cultured with omental cells in a 1∶1 ratio in complete RMPI 1640 media with or without 1 µg/ml anti-CD3 unless mentioned otherwise. In some experiments 0.1 mM L-NAME (Sigma, St. Louis, MO) was added. For some experiments, CD45 positive and negative fractions were sorted using the IMag Cell Separation System (BDbiosciences, San Jose, CA). Cell purity was >90%. Cells were harvested at the indicated time points and the total number of live cells were determined by trypan blue exclusion, and then labeled and analyzed on the FACSCanto flow cytometer (BD Biosciences, San Jose, CA).

For detection of iNOS expression, cells were cultured for 24 hours with or without 10 ng/ml recombinant IFNγ (Peprotech Inc., Rocky Hill, NJ).

Effector T cell differentiation was induced by stimulating CD4 T cells with anti-CD3 antibody with the following additives. **Th1**: anti-IL-4 (2 µg/ml), mrIL-12 (10 ng/ml); **Th2**: anti-IL-12 (2 µg/ml), IL-4 (10 ng/ml); **Th17**: mrIL-6 (20 ng/ml), mrIL-23 (10 ng/ml), hTGFβ (2.5 ng/ml), anti-IL-4 (2 µg/ml), anti-IFNγ (2 µg/ml); **iTreg**: hTGFβ (2.5 ng/ml), mrIL-2 (10 µg/ml), anti-IL-4 (2 µg/ml), anti-IFNγ (2 µg/ml). nTreg was expanded by the method described previously [71]. On 5th day of culture, omentum cells were added at a ratio of 1∶1, for a total of 2×10^6^ cells in differentiation media for 2 days. Then, Th1, Th2 and Th17 cells were stimulated with PMA/Ionomycin/monensin for 4 hours and applied for cytokine expression analysis by intracellular staining.

### Bleomycin induced lung injury model

All experiments were carried out in C57BL/6 mice weighing 22–25 g. Lung injury was induced by intratracheal administration of bleomycin. Mice anesthetized by inhalation of isoflurane inhalation (Hospira, Lake Forest, IL) were given an incision in the neck region to expose the trachea. 0.04 U bleomycin (Sigma-Aldrich Inc. St. Louis, MO) was instilled IT in 30 µl sterile isotonic saline as previously described [27]. The volume density of lesion was carried out as described previously [72]. Point counting techniques were used to estimate the volume density, VV, of lesion using the formula VV = PP = PN/PT where PP is the point fraction of PN, the number of test points hitting a lesion divided by PT, the total number of points hitting the section. A lesion was defined as a cluster of four or more inflammatory cells in either interstitium or airways. The slide was systematically scanned with a 49- point square lattice until all fields of the section were counted.

### Cell lysis and western blot

For western blot analysis, cells were lysed in Laemmli buffer (containing 4%SDS, 10% beta-mercaptoethanol, 20% glycerol, and 125 mM Tris, pH 6.8), boiled, and frozen at −20°C until analysis. Lysates from equal number of cells were loaded in each well, followed by electrophoresis and blotting onto PVDF membrane. Membranes were probed with antibodies specific for iNOS (BD Biosciences, San Jose, CA), and β-actin (Sigma-Aldrich Inc. St. Louis, MO), followed by the appropriate HRP-conjugated secondary antibodies (Cell signaling, Danver, MA) and SuperSignal West Pico or SuperSignal West Femto Substrate (Pierce, Rockford, IL).

### Lung and osteoblast differentiation culture media

Magnetic bead separation (IMag separation system) was performed on whole omentum isolating first those cells expressing CD45 from those not expressing, and a second separation was then performed on the CD45^−^ cells isolating those cells expressing CD34 from those not expressing CD34. Whole omentum cells and sorted omentum cells were placed into culture with either basal media or media enhanced to encourage differentiation into airway epithelial cells. Lung differentiation media was a succession of three different media formulations described previously [42] including media enhanced with activin A (R&D Systems, MN) and small airway growth media (Cell N Tec, Bern, Switzerland) as final inducing media. Medium inducing osteogenic differentiation consisted of Minimum Essential Medium, Fetal Bovine Serum, penicillin and streptotomycin, L-glutamine, and osteogenic supplements (R&D Systems) [44]. Cultures were maintained for five weeks at 37°C and 5% CO_2_ and then analyzed for expression of CCSP.

### Analysis of CCSP expression by RT-PCR

Total RNA was extracted and purified by μMACS mRNA isolation kit (Miltenyi Biotec, Bergisch Gladbach, Germany). First-strand cDNA was synthesized with random primers and reverse transcriptase (Promega, WI). CCSP and GAPDH were amplified by PCR (Initial denaturation at 95°C×5 mins, then 35 cycles of 95°×30 sec, 62°C×30 sec, 72°×30 sec, final extension with 72°×7 mins) with Taq polymerase. *Primer sequences*

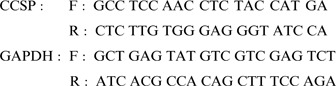



### Analysis of CCSP and Osteopontin expression by immunofluorescent staining

Omentum cells cultured on collagen-coated glass coverslips were fixed with 4% paraformaldehyde. Cells were permeabilized with 0.5% DAKO Tween 20 in PBS for 10 minutes followed by a series of washings. Samples were then blocked with 5% normal donkey serum, 0.5% BSA in PBS for 1 hour at RT. Goat anti-rat CCSP (courtesy of Barry Stripp, Duke University) at 1∶1000 dilution or Goat anti-mouse Osteopontin (R&D System) at 1∶200 dilution was applied and incubated overnight in the dark at 4°C in dilutent buffer. Following a series of washings with PBS the secondary antibody, donkey anti-goat Alexa 594 (Invitrogen) at 1∶250 dilution was applied and incubated for 2 hours at RT in the dark. Following a series of washings, the specimens were mounted with ProLong Gold antifade reagent with DAPI (Invitrogen) and analyzed with Zeiss confocal microscopy.

### Bronchoalveolar Lavage and Flow Cytometry

Animals were euthanized by injecting an overdose of pentobarbital. The abdominal vein was cut and the chest opened. The trachea was exposed and intubated with a 14 gauge catheter. Lungs were then lavaged with 5×1 ml ice-cold PBS and 4–4.5 ml were recovered. BAL was centrifuged and cells were counted using a Coulter Counter (Coulter Electornics, Hialeah, FL). The supernatant was either analyzed by ELISA or frozen for later analysis.

T cell subpopulations were analyzed by flow cytometry using a five-color staining protocol with CD4-Pacific orange/CD3-PE-Cy7/CD45-APC/CD8-APC-Cy5.5/γδ-Alexa750 and analyzed with a CyFlow ML (Partec Inc. NJ). Gates were set around the lymphocyte population and at least 3000 CD3 positive cells were analyzed. The total number of CD4, CD8, and γδ T cells was calculated by multiplying the total BAL cell number by the proportion of CD45 and CD3 and by the proportion of the analyzed subpopulation respectively.

### Statistical analysis

One-way ANOVA was used for statistical analysis of more than one group of samples. For all other data, unpaired t test was used. p value of <0.05 was considered statistically significant.

## Supporting Information

Figure S1 **Expansion of omentum in response to polyacrylamide bead injection into the peritoneal cavity of C57BL/6 mice.** Pictures of the omentum attached to the stomach from a naïve mouse and from a day 7 bead- injected mouse.
(TIF)

## References

[1] Cannaday JE (1948). Some uses of undetached omentum in surgery.. Am J Surg.

[2] Vineberg AM, Kato Y, Pirozynski WJ (1966). Experimental revascularization of the entire heart. Evaluation of epicardiectomy, omental graft, and/or implantation of the internal mammary artery in preventing myocardial necrosis and death of the animal.. Am Heart J.

[3] Vernik J, Singh AK (2007). Omentum: power to heal and regenerate.. Int J Artif Organs.

[4] Collins D, Hogan AM, O'Shea D, Winter DC (2009). The omentum: anatomical, metabolic, and surgical aspects.. J Gastrointest Surg.

[5] Liebermann-Meffert D (2000). The greater omentum. Anatomy, embryology, and surgical applications.. Surg Clin North Am 80: 275–293,.

[6] Athanassiadi K, Theakos N, Benakis G, Kakaris S, Skottis I (2007). Omental transposition: the final solution for major sternal wound infection.. Asian Cardiovasc Thorac Ann.

[7] Falagas ME, Rosmarakis ES (2006). Recurrent post-sternotomy mediastinitis.. J Infect.

[8] Moreschi AH, Macedo Neto AV, Barbosa GV, Saueressig MG (2008). Aggressive treatment using muscle flaps or omentopexy in infections of the sternum and anterior mediastinum following sternotomy.. J Bras Pneumol.

[9] Asai S, Kamei Y, Torii S (2004). One-stage reconstruction of infected cranial defects using a titanium mesh plate enclosed in an omental flap.. Ann Plast Surg.

[10] Goldsmith HS, Chen WF, Duckett SW (1973). Brain vascularization by intact omentum.. Arch Surg.

[11] Vatansev C, Ustun ME, Ogun CO, Tastekin G, Karabacakoglu A (2003). Omental transposition decreases ischemic brain damage examined in a new ischemia model.. Eur Surg Res.

[12] Maloney CT, Wages D, Upton J, Lee WP (2003). Free omental tissue transfer for extremity coverage and revascularization.. Plast Reconstr Surg.

[13] Patel RS, Gilbert RW (2009). Utility of the gastro-omental free flap in head and neck reconstruction.. Curr Opin Otolaryngol Head Neck Surg.

[14] Bayles SW, Hayden RE (2008). Gastro-omental free flap reconstruction of the head and neck.. Arch Facial Plast Surg.

[15] Goldsmith HS, de la Torre JC (1992). Axonal regeneration after spinal cord transection and reconstruction.. Brain Res.

[16] de la Torre JC, Goldsmith HS (1994). Coerulospinal fiber regeneration in transected feline spinal cord.. Brain Res Bull.

[17] Goldsmith HS (1994). Brain and spinal cord revascularization by omental transposition.. Neurol Res.

[18] Rogers SA, Chen F, Talcott M, Hammerman MR (2004). Islet cell engraftment and control of diabetes in rats after transplantation of pig pancreatic anlagen.. Am J Physiol Endocrinol Metab.

[19] Lee H, Cusick RA, Utsunomiya H, Ma PX, Langer R (2003). Effect of implantation site on hepatocytes heterotopically transplanted on biodegradable polymer scaffolds.. Tissue Eng.

[20] Sigrist S, Mechine-Neuville A, Mandes K, Calenda V, Legeay G (2003). Induction of angiogenesis in omentum with vascular endothelial growth factor: influence on the viability of encapsulated rat pancreatic islets during transplantation.. J Vasc Res.

[21] Kin T, Korbutt GS, Rajotte RV (2003). Survival and metabolic function of syngeneic rat islet grafts transplanted in the omental pouch.. Am J Transplant.

[22] Hammerman MR (2004). Renal organogenesis from transplanted metanephric primordia.. J Am Soc Nephrol.

[23] Litbarg NO, Gudehithlu KP, Sethupathi P, Arruda JA, Dunea G (2007). Activated omentum becomes rich in factors that promote healing and tissue regeneration.. Cell Tissue Res.

[24] Singh AK, Patel J, Litbarg NO, Gudehithlu KP, Sethupathi P (2008). Stromal cells cultured from omentum express pluripotent markers, produce high amounts of VEGF, and engraft to injured sites.. Cell Tissue Res.

[25] Singh AK, Pancholi N, Patel J, Litbarg NO, Gudehithlu KP (2009). Omentum facilitates liver regeneration.. World J Gastroenterol.

[26] Garcia-Gomez I, Goldsmith HS, Angulo J, Prados A, Lopez-Hervas P (2005). Angiogenic capacity of human omental stem cells.. NeurolRes.

[27] Braun RK, Ferrick C, Neubauer P, Sjoding M, Sterner-Kock A (2008). IL-17 producing gammadelta T cells are required for a controlled inflammatory response after bleomycin-induced lung injury.. Inflammation.

[28] Avouac J, Furnrohr BG, Tomcik M, Palumbo K, Zerr P, et al. Inactivation of the transcription factor STAT-4 prevents inflammation-driven fibrosis in animal models of systemic sclerosis.. Arthritis Rheum.

[29] Liu T, Baek HA, Yu H, Lee HJ, Park BH (2011). FIZZ2/RELM-beta induction and role in pulmonary fibrosis.. J Immunol.

[30] Oh K, Park HB, Byoun OJ, Shin DM, Jeong EM (2011). Epithelial transglutaminase 2 is needed for T cell interleukin-17 production and subsequent pulmonary inflammation and fibrosis in bleomycin-treated mice.. J Exp Med.

[31] Pond CM (2003). Paracrine relationships between adipose and lymphoid tissues: implications for the mechanism of HIV-associated adipose redistribution syndrome.. Trends Immunol.

[32] Peister A, Mellad JA, Larson BL, Hall BM, Gibson LF (2004). Adult stem cells from bone marrow (MSCs) isolated from different strains of inbred mice vary in surface epitopes, rates of proliferation, and differentiation potential.. Blood.

[33] Peranzoni E, Zilio S, Marigo I, Dolcetti L, Zanovello P (2010). Myeloid-derived suppressor cell heterogeneity and subset definition.. Curr Opin Immunol.

[34] Gabrilovich DI, Nagaraj S (2009). Myeloid-derived suppressor cells as regulators of the immune system.. Nat Rev Immunol.

[35] Ugel S, Delpozzo F, Desantis G, Papalini F, Simonato F (2009). Therapeutic targeting of myeloid-derived suppressor cells.. Curr Opin Pharmacol.

[36] Mazzoni A, Bronte V, Visintin A, Spitzer JH, Apolloni E (2002). Myeloid suppressor lines inhibit T cell responses by an NO-dependent mechanism.. J Immunol.

[37] Ostrand-Rosenberg S, Sinha P (2009). Myeloid-derived suppressor cells: linking inflammation and cancer.. J Immunol.

[38] Burlingham WJ, Love RB, Jankowska-Gan E, Haynes LD, Xu Q (2007). IL-17-dependent cellular immunity to collagen type V predisposes to obliterative bronchiolitis in human lung transplants.. Journal of Clinical Investigation.

[39] Rajesh RV, Heo GN, Park MR, Nam JS, Kim NK, et al. Proteomic analysis of bovine omental, subcutaneous and intramuscular preadipocytes during in vitro adipogenic differentiation.. Comp Biochem Physiol Part D Genomics Proteomics.

[40] Pancholi N, Patel J, Gudehithlu KP, Kraus MA, Dunea G, et al. Culture of omentum-induced regenerating liver yielded hepatocyte-committed stem cells.. Transl Res.

[41] Mohammadi R, Azizi S, Delirezh N, Hobbenaghi R, Amini K Comparison of beneficial effects of undifferentiated cultured bone marrow stromal cells and omental adipose-derived nucleated cell fractions on sciatic nerve regeneration.. Muscle Nerve.

[42] Rippon HJ, Polak JM, Qin M, Bishop AE (2006). Derivation of distal lung epithelial progenitors from murine embryonic stem cells using a novel three-step differentiation protocol.. Stem Cells.

[43] Sueblinvong V, Loi R, Eisenhauer PL, Bernstein IM, Suratt BT (2008). Derivation of lung epithelium from human cord blood-derived mesenchymal stem cells.. Am J Respir Crit Care Med.

[44] Wang W, Itaka K, Ohba S, Nishiyama N, Chung UI (2009). 3D spheroid culture system on micropatterned substrates for improved differentiation efficiency of multipotent mesenchymal stem cells.. Biomaterials.

[45] Cao YA, Wagers AJ, Beilhack A, Dusich J, Bachmann MH (2004). Shifting foci of hematopoiesis during reconstitution from single stem cells.. Proc Natl Acad Sci U S A.

[46] Morison R (1906). Remarks ON SOME FUNCTIONS OF THE OMENTUM.. Br Med J.

[47] Krist LF, Eestermans IL, Steenbergen JJ, Hoefsmit EC, Cuesta MA (1995). Cellular composition of milky spots in the human greater omentum: an immunochemical and ultrastructural study.. Anat Rec.

[48] Ray P, Arora M, Poe SL, Ray A (2011). Lung myeloid-derived suppressor cells and regulation of inflammation.. Immunol Res.

[49] Friedenstein AJ, Petrakova KV, Kurolesova AI, Frolova GP (1968). Heterotopic of bone marrow. Analysis of precursor cells for osteogenic and hematopoietic tissues.. Transplantation.

[50] Dominici M, Le Blanc K, Mueller I, Slaper-Cortenbach I, Marini F (2006). Minimal criteria for defining multipotent mesenchymal stromal cells. The International Society for Cellular Therapy position statement.. Cytotherapy.

[51] Makino S, Fukuda K, Miyoshi S, Konishi F, Kodama H (1999). Cardiomyocytes can be generated from marrow stromal cells in vitro.. J Clin Invest.

[52] Toma C, Pittenger MF, Cahill KS, Byrne BJ, Kessler PD (2002). Human mesenchymal stem cells differentiate to a cardiomyocyte phenotype in the adult murine heart.. Circulation.

[53] Wang G, Bunnell BA, Painter RG, Quiniones BC, Tom S (2005). Adult stem cells from bone marrow stroma differentiate into airway epithelial cells: potential therapy for cystic fibrosis.. Proc Natl Acad Sci U S A.

[54] Weiss DJ, Berberich MA, Borok Z, Gail DB, Kolls JK (2006). Adult stem cells, lung biology, and lung disease. NHLBI/Cystic Fibrosis Foundation Workshop.. Proc Am Thorac Soc.

[55] Spees JL, Olson SD, Ylostalo J, Lynch PJ, Smith J (2003). Differentiation, cell fusion, and nuclear fusion during ex vivo repair of epithelium by human adult stem cells from bone marrow stroma.. Proc Natl Acad Sci U S A.

[56] Schwartz RE, Reyes M, Koodie L, Jiang Y, Blackstad M (2002). Multipotent adult progenitor cells from bone marrow differentiate into functional hepatocyte-like cells.. J Clin Invest.

[57] Sato Y, Araki H, Kato J, Nakamura K, Kawano Y (2005). Human mesenchymal stem cells xenografted directly to rat liver are differentiated into human hepatocytes without fusion.. Blood.

[58] Sanchez-Ramos J, Song S, Cardozo-Pelaez F, Hazzi C, Stedeford T (2000). Adult bone marrow stromal cells differentiate into neural cells in vitro.. Exp Neurol.

[59] Munoz JR, Stoutenger BR, Robinson AP, Spees JL, Prockop DJ (2005). Human stem/progenitor cells from bone marrow promote neurogenesis of endogenous neural stem cells in the hippocampus of mice.. Proc Natl Acad Sci U S A.

[60] Moriscot C, de Fraipont F, Richard MJ, Marchand M, Savatier P (2005). Human bone marrow mesenchymal stem cells can express insulin and key transcription factors of the endocrine pancreas developmental pathway upon genetic and/or microenvironmental manipulation in vitro.. Stem Cells.

[61] Oh SH, Muzzonigro TM, Bae SH, LaPlante JM, Hatch HM (2004). Adult bone marrow-derived cells trans-differentiating into insulin-producing cells for the treatment of type I diabetes.. Lab Invest.

[62] Zuk PA, Zhu M, Mizuno H, Huang J, Futrell JW (2001). Multilineage cells from human adipose tissue: implications for cell-based therapies.. Tissue Eng.

[63] Salem HK, Thiemermann C (2010). Mesenchymal stromal cells: current understanding and clinical status.. Stem Cells.

[64] Hocking AM, Gibran NS (2010). Mesenchymal stem cells: paracrine signaling and differentiation during cutaneous wound repair.. Exp Cell Res.

[65] Bronte V (2009). Myeloid-derived suppressor cells in inflammation: uncovering cell subsets with enhanced immunosuppressive functions.. Eur J Immunol.

[66] Augello A, Kurth TB, De Bari C (2010). Mesenchymal stem cells: a perspective from in vitro cultures to in vivo migration and niches.. Eur Cell Mater.

[67] Gin H, Dupuy B, Bonnemaison-Bourignon D, Bordenave L, Bareille R (1990). Biocompatibility of polyacrylamide microcapsules implanted in peritoneal cavity or spleen of the rat. Effect on various inflammatory reactions in vitro.. Biomater Artif Cells Artif Organs.

[68] Christensen LH, Nielsen JB, Mouritsen L, Sorensen M, Lose G (2008). Tissue integration of polyacrylamide hydrogel: an experimental study of periurethral, perivesical, and mammary gland tissue in the pig.. Dermatol Surg 34 Suppl 1: S68–77; discussion.

[69] Crabtree JH, Fishman A (1999). Laparoscopic omentectomy for peritoneal dialysis catheter flow obstruction: a case report and review of the literature.. Surg Laparosc Endosc Percutan Tech.

[70] Garcia-Gomez I, Goldsmith HS, Angulo J, Prados A, Lopez-Hervas P (2005). Angiogenic capacity of human omental stem cells.. Neurol Res.

[71] Singh N, Yamamoto M, Takami M, Seki Y, Takezaki M (2010). CD4(+)CD25(+) regulatory T cells resist a novel form of CD28− and Fas-dependent p53-induced T cell apoptosis.. J Immunol.

[72] Nakashima JM, Hyde DM, Giri SN (1986). Effects of a calmodulin inhibitor on bleomycin-induced lung inflammation in hamsters. Biochemical, morphometric, and bronchoalveolar lavage data.. Am J Pathol.

