# Supplementary figures and images for: Cellular Basis of Tissue Regeneration by Omentum
Source: PLoS One. 2012 Jun 6;7(6):e38368. doi: 10.1371/journal.pone.0038368 (PMC3368844; doi:10.1371/journal.pone.0038368)

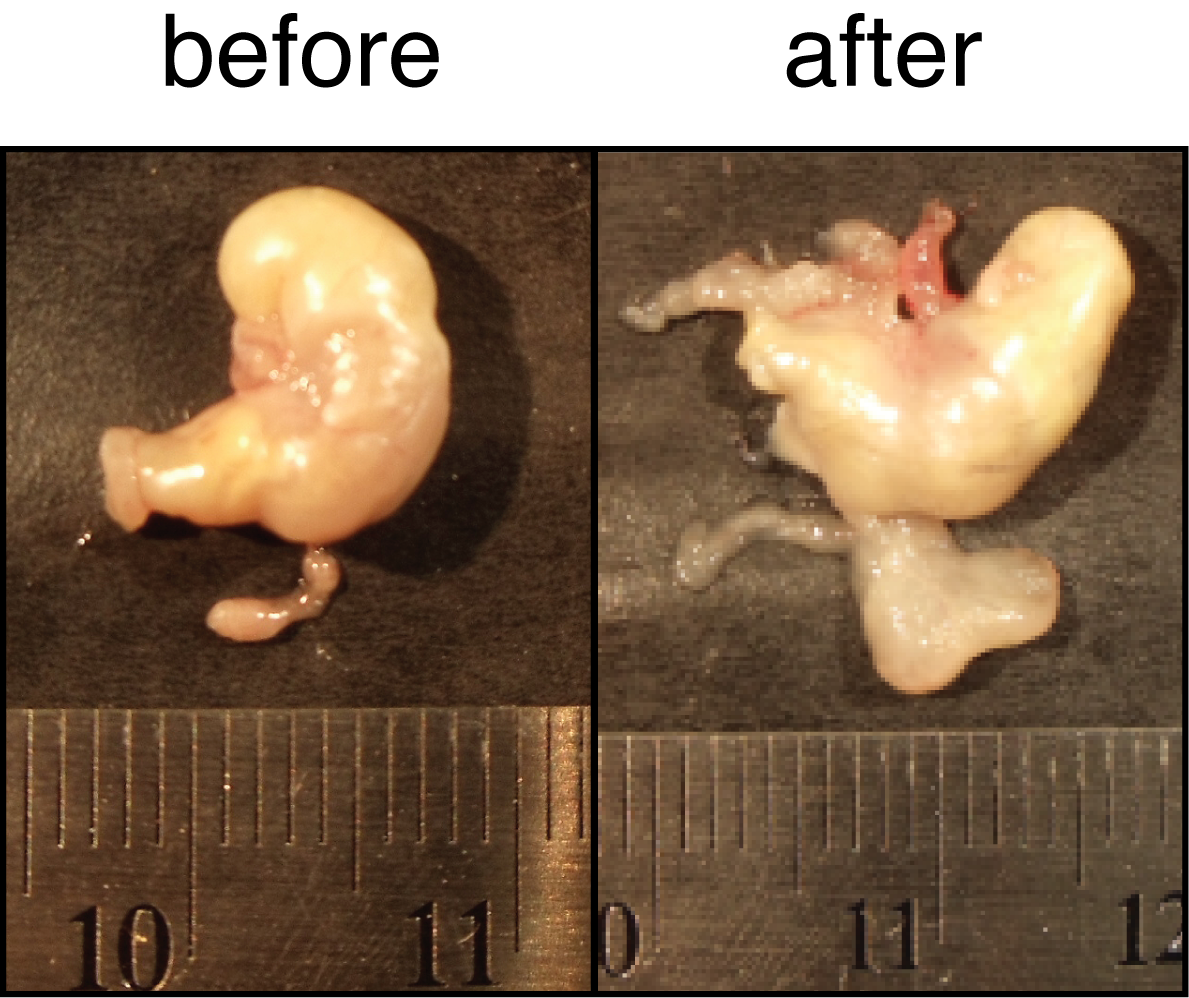

Supplement: Figure S1 — Expansion of omentum in response to polyacrylamide bead injection into the peritoneal cavity of C57BL/6 mice. Pictures of the omentum attached to the stomach from a naïve mouse and from a day 7 bead- injected mouse. (TIF) [file pone.0038368.s001.tif]
